# Exosomal microRNAs miR-30d-5p and miR-126a-5p Are Associated with Heart Failure with Preserved Ejection Fraction in STZ-Induced Type 1 Diabetic Rats

**DOI:** 10.3390/ijms23147514

**Published:** 2022-07-06

**Authors:** Jiung-Pang Huang, Chih-Chun Chang, Chao-Yu Kuo, Kuang-Jing Huang, Etienne M. Sokal, Kuan-Hsing Chen, Li-Man Hung

**Affiliations:** 1 Department and Graduate Institute of Biomedical Sciences, College of Medicine, Chang Gung University, Taoyuan 333, Taiwan; dddivekimo@yahoo.com.tw (J.-P.H.); as22340@hotmail.com (C.-Y.K.); 2 Healthy Aging Research Center, Chang Gung University, Taoyuan 333, Taiwan; 3 Department of Clinical Pathology, Far Eastern Memorial Hospital, New Taipei 220, Taiwan; chihchun.chang1211@gmail.com; 4 Graduate Institute of Clinical Medicine Science, College of Medicine, Chang Gung University, Taoyuan 333, Taiwan; 5 Department of Nursing, Cardinal Tien Junior College of Healthcare and Management, Yilan 266, Taiwan; 6 Microscopy Center, Chang Gung University, Taoyuan 333, Taiwan; hkjeason@gmail.com; 7 Molecular Medicine Research Center, Chang Gung University, Taoyuan 333, Taiwan; 8 Laboratory of Pediatric Hepatology and Cell Therapy, Institut de Recherche Expérimentale et Clinique (IREC), Université Catholique de Louvain, 1200 Brussels, Belgium; sokal@saintluc.uclouvain.be; 9 Kidney Research Center, Chang Gung Memorial Hospital, Linkou 333, Taiwan; guanhsing@yahoo.com.tw

**Keywords:** diabetes, HFpEF, exosome, miRNA, biomarker

## Abstract

Exosomal microRNAs (EXO-miRNAs) are promising non-invasive diagnostic biomarkers for cardiovascular disease. Heart failure with preserved ejection fraction (HFpEF) is a poorly understood cardiovascular complication of diabetes mellitus (DM). Little is known about whether EXO-miRNAs can be used as biomarkers for HFpEF in DM. We aimed to investigate the relationship between EXO-miRNAs and HFpEF in STZ-induced diabetic rats. We prepared STZ-induced diabetic rats exhibiting a type 1 DM phenotype with low body weight, hyperglycemia, hyperlipidemia and hypoinsulinemia. Histological sections confirmed atrophy and fibrosis of the heart, with collagen accumulation representing diabetic cardiomyopathy. Significant decreases in end-diastolic volume, stroke volume, stroke work, end-systolic elastance and cardiac output indicated impaired cardiac contractility, as well as mRNA conversion of two isoforms of myosin heavy chain (α-MHC and β-MHC) and increased atrial natriuretic factor (ANF) mRNA indicating heart failure, were consistent with the features of HFpEF. In diabetic HFpEF rats, we examined a selected panel of 12 circulating miRNAs associated with HF (miR-1-3p, miR-21-5p, miR-29a-5p, miR-30d-5p, miR-34a-5p, miR-126a-5p, miR-143-3p, miR-145-5p, miR-195-5p, miR-206-3p, miR-320-3p and miR-378-3p). Although they were all expressed at significantly lower levels in the heart compared to non-diabetic controls, only six miRNAs (miR-21-5p, miR-30d-5p, miR-126a-5p, miR-206-3p, miR-320-3p and miR-378-3p) were also reduced in exosomal content, while one miRNA (miR-34a-5p) was upregulated. Similarly, although all miRNAs were correlated with reduced cardiac output as a measure of cardiovascular performance, only three miRNAs (miR-30d-5p, miR-126a-5p and miR-378-3p) were correlated in exosomal content. We found that miR-30d-5p and miR-126a-5p remained consistently correlated with significant reductions in exosomal expression, cardiac expression and cardiac output. Our findings support their release from the heart and association with diabetic HFpEF. We propose that these two EXO-miRNAs may be important for the development of diagnostic tools for diabetic HFpEF.

## 1. Introduction

Heart failure (HF) is a major cardiovascular complication of diabetes mellitus (DM) and can be classified as systolic heart failure with reduced ejection fraction (EF < 40%, HFrEF), which occurs when the heart beats too weakly to circulate blood throughout the body, or diastolic heart failure with preserved ejection fraction (HFpEF), which occurs when the left ventricle loses its ability to relax normally and fills less blood at rest [1,2,3]. HFrEF is generally preceded by acute or chronic loss of cardiomyocytes due to ischemia injury, myocarditis, genetic mutations, valvular disease, lipotoxicity, advanced glycation end products (AGEs)-induced cardiomyocyte death and replacement fibrosis, whereas HFpEF is preceded by chronic comorbidities, such as obesity and DM, involving microvascular endothelial inflammation and cardiac fibrosis [4,5].

Defining HFpEF is much more complex because LVEF is preserved and the diagnosis requires clinical symptoms and/or heart failure and other evidence such as structural heart disease (cardiac fibrosis) or diastolic dysfunction [6]. To date, the progression of cardiomyopathy is associated with cardiac expression of natriuretic peptides (NPs), and elevated NP levels are used to help diagnose HFpEF [7,8]. However, the pathophysiology of HFpEF is still poorly understood. Currently available treatments have improved the condition of patients with HFrEF, but not in patients with HFpEF [9].

Cardiac-specific miRNAs are involved in cardiac function and in the development of cardiac diseases such as HF, fibrosis, hypertrophy and maladaptation [10,11,12]. It is widely accepted that identifying circulating miRNA is useful for predicting HF [13]. MiRNAs have been demonstrated in HF [14,15], and they are short, non-coding RNA molecules that negatively affect post-transcriptional regulation. For example, miR-24, miR-34, miR-126 and miR-214 exert anti-angiogenic effects [14]. MiR-1, miR-133, miR-155, miR-185 and miR-378 reduce cardiac hypertrophy [14]. MiR-24, miR-26a, miR-29 and miR-133 exhibit anti-fibrotic effects [14]. Thus, miRNAs are considered potential markers and therapeutic targets for HF.

Exosomal miRNAs (EXO-miRNAs) provide a more stable miRNA resource than circulating miRNAs. Exosome-encapsulated miRNAs are protected by membranes that resist degradation [16]. Exosomes (EXO) are one of the small extracellular vesicles that are produced by cell membrane shedding or internal compartments [17,18]. They are involved in antigen presentation, cellular transformation and intercellular communication by delivering proteins, lipids and genetic material (e.g., DNA, RNA and miRNA) to recipient cells along with bioactive molecules [19,20,21]. In this study, we hypothesized that miRNAs play a pathophysiological role in the progression of diabetic HFpEF and the associated EXO-miRNAs can be reliably detected. Studying diabetic HFpEF allows us to understand its mechanistic progression and to develop biomarkers and therapeutic approaches for diabetic HFpEF.

## 2. Results

### 2.1. STZ-Induced T1DM

**Figure 1:**
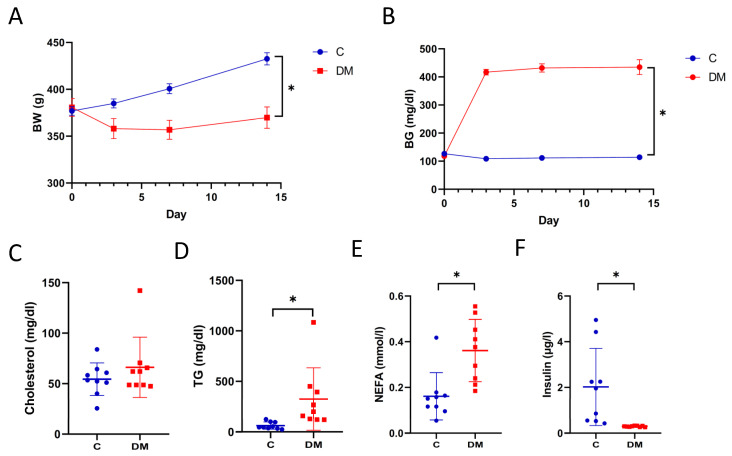
STZ-induced T1DM. (**A**) Body weight—BW. (**B**) Blood glucose—BG. (**C**) Plasma cholesterol levels. (**D**) Plasma TG levels. (**E**) Plasma non-esterified fatty acids, NEFA levels. (**F**) Plasma insulin levels. Data are expressed as mean ± SEM (*n* = 9). *p*-values were determined by Student’s *t*-test. * *p* < 0.05 DM vs. C.

Rodent models have been used to study diabetic HFpEF. STZ administration is a reliable model for studying type 1 diabetes mellitus (T1DM). STZ-induced diastolic dysfunction with preserved EF provides a viable model for HFpEF studies [22,23]. Body weight (BW) was decreased in diabetic rats compared to controls (C) (Figure 1A). Hyperglycemia was observed at all observation points (days 3, 7 and 14) after STZ administration (Figure 1B). STZ increased plasma TG (Figure 1D) and non-esterified fatty acids (NEFA) (Figure 1E) while decreased plasma insulin levels (Figure 1F), overall indicating a T1DM phenotype.

### 2.2. STZ-Induced Diabetic Cardiomyopathy and Cardiac Fibrosis

**Figure 2:**
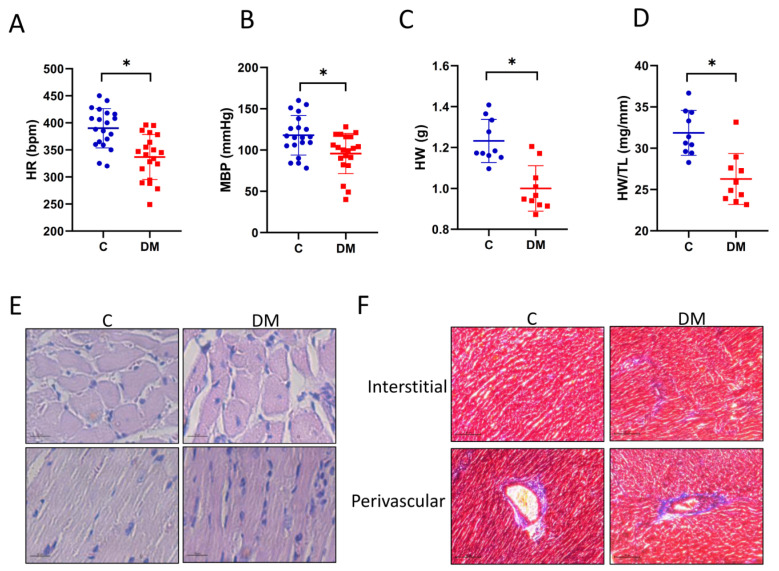

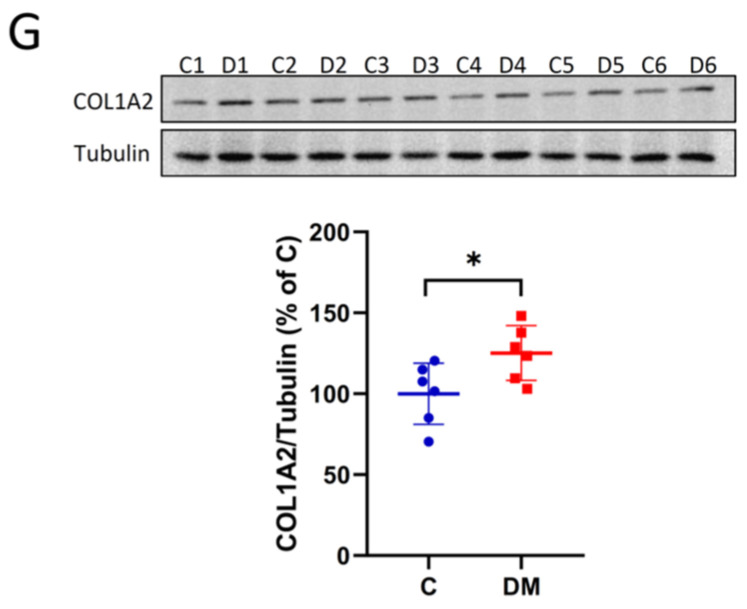
STZ-induced diabetic cardiomyopathy and cardiac fibrosis. (**A**) Heart rate—HR, *n* = 20. (**B**) Mean blood pressure—MBP, *n* = 20. (**C**) Heart weight—HW, *n* = 10. (**D**) Ratio of heart weight/tibial length—HW/TL, *n* = 10. Data are expressed as mean ± SEM. *p*-values were determined by Student‘s *t*-test. (**E**) Images of individual cardiomyocytes (scale bar = 20 μm). (**F**) Masson trichrome staining to detect fibrosis in interstitial (top) and perivascular (bottom) tissue (scale bars = 100 μm). (**G**) Cardiac COL1A2 protein levels and their quantitative results, *n* = 6. Data are expressed as mean ± SEM. *p*-values were determined by Student’s *t*-test. * *p* < 0.05 DM vs. C.

Cardiac morphology was assessed to determine structural changes in T1DM animals. Heart rate (HR) (Figure 2A) and mean blood pressure (MBP) (Figure 2B) were decreased. STZ administration decreased heart weight (HW) (Figure 2C), as well as HW/TL (HW normalized by tibia length, TL) (Figure 2D). Cardiomyocyte morphology was not significantly altered in DM hearts (Figure 2E). One of the hallmarks of HFpEF is cardiac fibrosis accompanied by collagen accumulation. As shown in Figure 2F,G, marked interstitial and perivascular fibrosis, as well as an increase in collagen type I alpha 2 chain (COL1A2), was observed in DM hearts. In summary, STZ administration induced fibrotic cardiomyopathy.

### 2.3. STZ-Induced HFpEF

**Figure 3:**
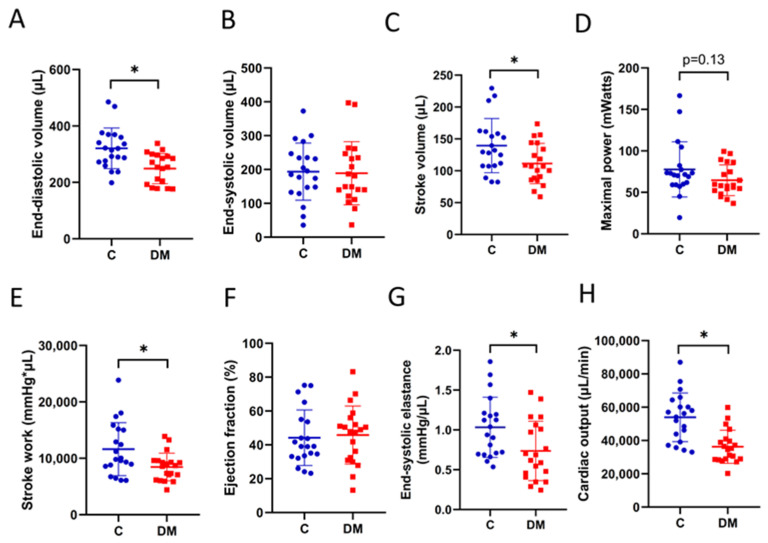
STZ-induced HFpEF. Cardiac hemodynamics were examined using cardiac P-V catheterization. (**A**) End-diastolic volume. (**B**) End-systolic volume. (**C**) Stroke volume. (**D**) Maximal power. (**E**) Stroke work. (**F**) Ejection fraction. (**G**) End-systolic elastance. (**H**) Cardiac output. Data are expressed as mean ± SEM (*n* = 20). *p*-values were determined by Student’s *t*-test. * *p* < 0.05 DM vs. C.

To classify STZ-induced diabetic cardiomyopathy, changes in hemodynamics and cardiac contractility were assessed using a P–V catheter, as described elsewhere [24]. A reduction in end-diastolic volume and comparable end-systolic volume was observed (Figure 3A,B), along with a reduction in stroke volume in the diabetic heart (Figure 3C). The reduction in stroke volume and stroke work indicated impaired contractility (Figure 3C,E). The ejection fraction remained unchanged in DM compared to controls (Figure 3F). End-systolic elastance and cardiac output were reduced in the diabetic heart (Figure 3G,H). Together, these cardiac hemodynamic indices demonstrate the HFpEF phenotype.

**Figure 4:**
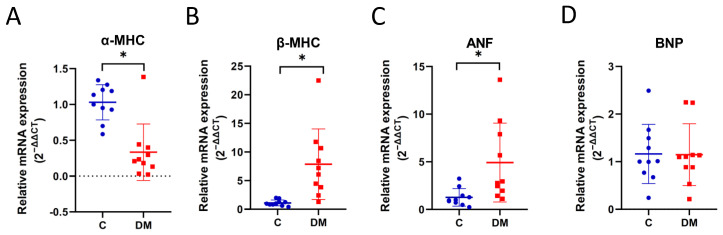
Changes in MHC and ANF mRNA in diabetic HFpEF hearts. Expression of mRNA was examined using RT-qPCR. (**A**) α-MHC, (**B**) β-MHC, (**C**) ANF and (**D**) BNP mRNA levels in the heart. Data are expressed as mean ± SEM (*n* = 10). *p*-values were determined by Student’s *t*-test. * *p* < 0.05 DM vs. C.

Conversion of 2 isoforms of myosin heavy chain (α-MHC and β-MHC) has been shown in patients with HF [25], and ANF is a key indicator for the diagnosis of HFpEF. From our experiments, mRNA levels of α-MHC decreased, and mRNA levels of β-MHC increased in the diabetic hearts (Figure 4A,B). The mRNA levels of ANF increased (Figure 4C), while mRNA levels of BNP did not differ between controls and T1DM (Figure 4D). These results suggest that STZ-induced diabetic cardiomyopathy can be classified as HFpEF.

### 2.4. Diabetic HF-Associated miRNA Expression in STZ-Induced HFpEF Hearts

**Figure 5:**
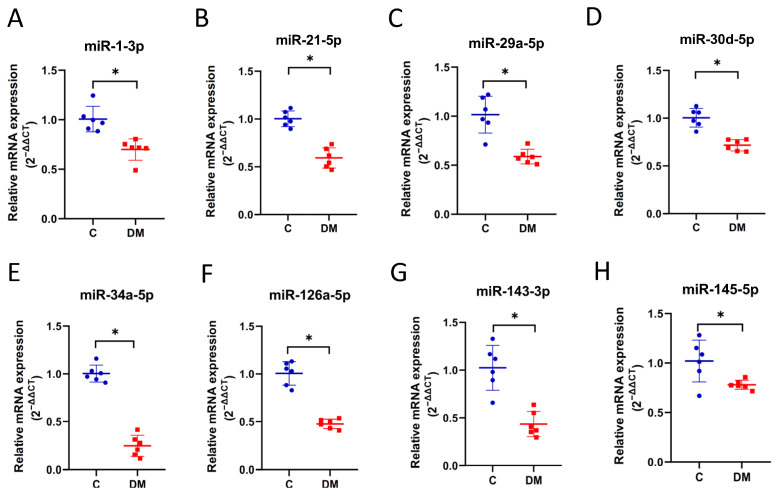

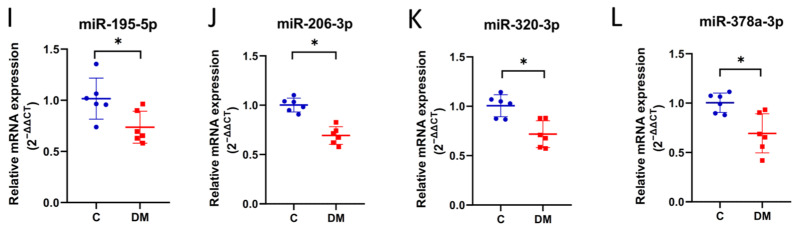
Expression of miRNAs in diabetic HFpEF hearts. The levels of miRNAs were examined using stem-loop RT-qPCR. (**A**) miR-1-3p. (**B**) miR-21-5p. (**C**) miR-29a-5p. (**D**) miR-30d-5p. (**E**) miR-34a-5p. (**F**) miR-126-5p. (**G**) miR-143-3p. (**H**) miR-145-5p. (**I**) miR-195-5p. (**J**) miR-206-3p. (**K**) miR-320-3p. (**L**) miR-378-3p. Data are expressed as mean ± SEM (*n* = 6). *p*-values were determined by Student’s *t*-test. * *p* < 0.05 DM vs. C.

Many tissue and circulating miRNAs are involved in the development of HF in patients [26], suggesting that miRNAs can be used for HF diagnosis and HF subtype classification [15,27]. We selected 12 miRNAs associated with diabetic HF. The initial selection of miRNAs in our panel were miR-1 [28,29], miR-21 [30], miR-29a [31], miR-30d [32,33], miR-34a [34], miR-126a [35], miR-143 [36], miR-145 [37], miR-195 [38], miR-206 [39], miR-320 [39] and miR-378 [40]. We found that the expression of all miRNAs was significantly lower in HFpEF diabetic hearts than in controls (Figure 5A–L). Furthermore, the expression levels of all miRNAs were positively correlated with reduced cardiac output, with miR-378-3p showing marginal significance (*p*-value = 0.057) (Table 1). Pearson correlation showed correlations ranging from moderate to very strong (Table 1).

### 2.5. EXO-miRNAs Associated with Cardiac Output and Cardiac Expression

**Figure 6:**
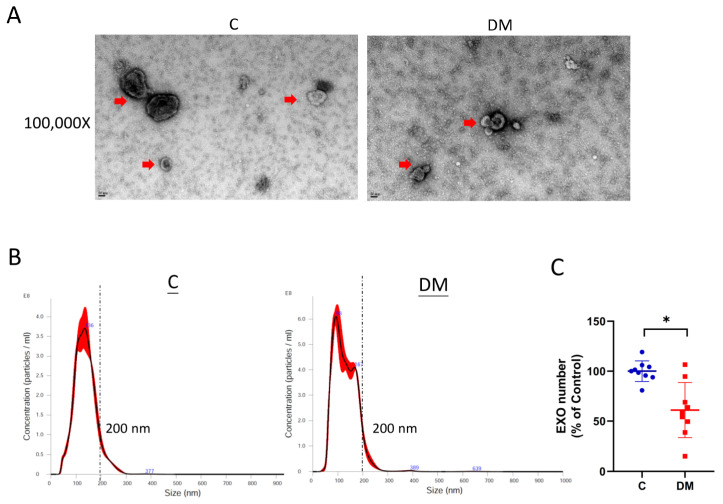
Characterization of plasma EXO in control and DM rats. (**A**) Photographs of EXO taken using transmission electron microscopy (TEM). EXO is shown at a magnification of 100,000X (scale bar = 50 nm). The red arrow indicates the isolated EXO. (**B**) Size-concentration distribution of EXO. (**C**) Reduced number of EXO in T1DM rats. Data are expressed as mean ± SEM (*n* = 9). *p*-values were determined by Student’s *t*-test. * *p* < 0.05 DM vs. C.

**Figure 7:**
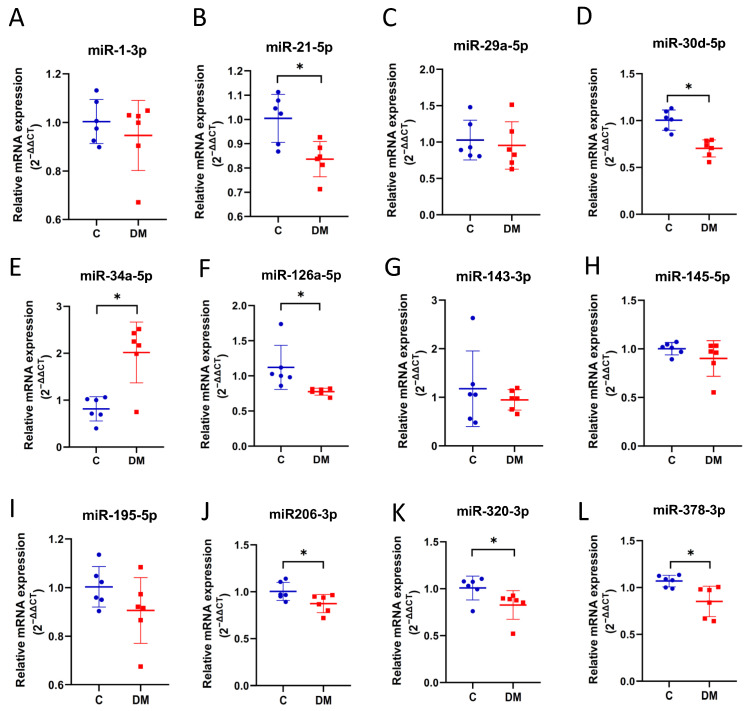
Expression of miRNAs in plasma EXO. The levels of miRNAs were examined using stem-loop RT-qPCR. (**A**) miR-1-3p. (**B**) miR-21-5p. (**C**) miR-29a-5p. (**D**) miR-30d-5p. (**E**) miR-34a-5p. (**F**) miR-126-5p. (**G**) miR-143-3p. (**H**) miR-145-5p. (**I**) miR-195-5p. (**J**) miR-206-3p. (**K**) miR-320-3p. (**L**) miR-378-3p. Data are expressed as mean ± SEM (*n* = 6). *p*-values were determined by Student’s *t*-test. * *p* < 0.05 DM vs. C.

EXO-encapsulated miRNAs provide a more stable source for addressing the role of HF-associated miRNAs in diabetic HFpEF. As shown in Figure 6A–C, plasma EXO was isolated and characterized. A smaller EXO population was observed in T1DM rats (Figure 6B and Figure S1). Reduced EXO content indicated impaired EXO production in T1DM animals (Figure 6C and Figure S2). The EXO content of six miRNAs (miR-21-5p, miR-30d-5p, miR-126a-5p, miR-206-3p, miR-320-3p and miR-378-3p) was decreased (Figure 7B,D,F,J–L), while miR-34a-5p was upregulated (Figure 7E). The levels of the remaining five miRNAs (miR-1-3p, miR-29a-5p, miR-143-3p, miR-145-5p and miR-195-5p) were comparable in DM rats and controls (Figure 7A,C,G–I). In addition, three EXO-miRNAs (miR-30d-5p, miR-126a-5p and miR-378-3p) were correlated with reduced cardiac output in diabetic HFpEF. Pearson correlation showed correlations were strong (Table 2).

**Figure 8:**
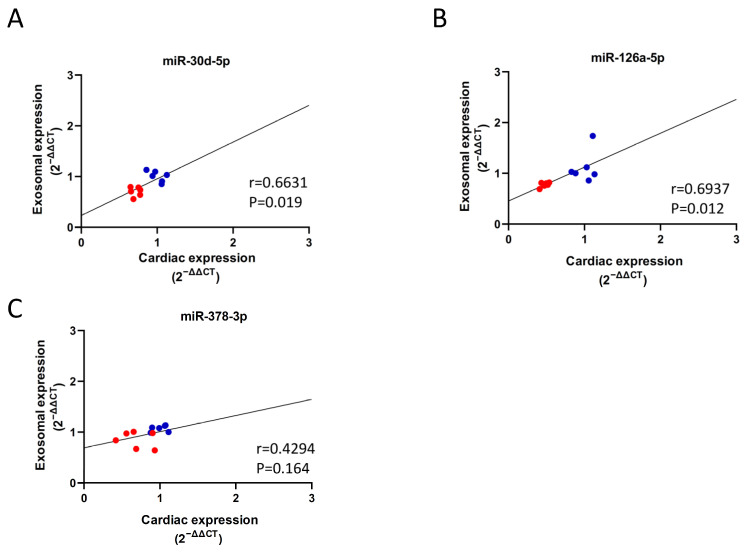
Correlation between exosomal expression and cardiac expression of miRNAs in diabetic HFpEF hearts. Pearson correlation coefficient (r) was used to determine the relationship between exosomal and cardiac expression in 12 total samples; control *n* = 6 (blue) and DM *n* = 6 (red). (**A**) miR-30d-5p shows a strong positive correlation. (**B**) miR-126a-5p shows a strong positive correlation. (**C**) miR-378-3p shows a moderate positive correlation.

Because the expression levels of these three miRNAs strongly correlated with cardiac output and EXO content in the same direction, we further tested their levels in the heart to observe the correlation between EXO and heart. Only miR-30d and miR126a showed a significant strong positive correlation (Figure 8A,B), while miR-378 showed a moderate but insignificant positive correlation (Figure 8C).

## 3. Discussion

In this study, we investigated the expression of HF-associated miRNAs in HFpEF diabetic rats. We established HFpEF hearts in STZ-induced T1DM rats exhibiting significant physical (lower BW and higher BG), biochemical (increased TG and NEFA and reduced insulin) and physiological (reduced EDV, stroke volume, stroke work, end systolic elastance and cardiac output) changes, as well as cardiac fibrosis, conversion of α-MHC and β-MHC, and increased ANF mRNA levels. Based on this rodent model, we observed reduced expression of 12 HF-associated miRNAs in HFpEF hearts compared to non-diabetic controls (Figure 5), six of which were downregulated in EXO content, and one (miR-34a) was upregulated in EXO content (Figure 7). We examined the relationship between miRNAs and cardiac output as a measure of abnormal diastolic function in HFpEF. All miRNAs were significantly correlated with reduced cardiac output (Table 1), three of which were correlated in EXO content (miR-30d-5p, miR-126a-5p and miR-378-3p) (Table 2). Finally, two (miR-30d-5p and miR-126a-5p) remained correlated with reduced exosomal expression, cardiac expression and cardiac output in HFpEF diabetic rats compared to non-diabetic rats (Figure 8A,B).

HFpEF animal models have been proposed, and it is important to ensure that animal models exhibit key features of HFpEF before conducting preclinical studies [23,41]. Roh et al. illustrated the feasibility of a phenotype-based stepwise approach to studying HFpEF models [41]. STZ-induced T1DM is a widely accepted animal model of diabetic cardiovascular disease and cardiac dysfunction [42,43]. Our STZ-T1DM rats demonstrate many features of HFpEF. The decrease in CO (Figure 3H) and the increase in cardiac ANP mRNA expression (Figure 4C) fulfilled the first criterion, i.e., confirmation of heart failure. Second, preservation of EF was observed (Figure 3G). The reduction in end diastolic volume in STZ rats (Figure 3A) fulfilled the third step, i.e., LV diastolic dysfunction. Although extracardiac parameters (step 4) were not determined in our work, vascular stiffness [44], skeletal muscle atrophy [45] and renal dysfunction [46] have been shown elsewhere. Thus, the STZ-induced rodent model provides us with a useful tool to study diabetic HFpEF.

Our findings support the association of miR-30d-5p and miR-126a-5p with diabetic HFpEF. The clinical significance of our results is that exosomal miR-30d and miR126a may be useful biomarkers and therapeutic targets for HFpEF in diabetic patients. Different miRNA expression patterns have been found to be associated with various pathophysiological mechanisms of HF, such as hypoxia, apoptosis, hypertrophy and cardiac remodeling [15]. It has been shown that circulating miR-30d or its family members (miR-30b and miR-30d) are downregulated in HF, and this downregulation reduces the cardioprotective role of miR-30d in HF [47,48,49]. MiR-126a reduction is a potential biomarker for DM and contributes to heart failure [50,51]. MiR-126a downregulation decreases cardiac microvessel density and impairs ventricular function [51]. How miR-30d and miR-126a downregulation leads to HFpEF is unclear, and we suggest that it may reduce cardioprotection and stimulate pathological remodeling in our STZ rodents under stress. More advanced studies are necessary to determine the underlying details of miR-30d and miR-126a in HFpEF. We also found that miR-34a-5p was downregulated in DM-heart tissue but upregulated in DM-exosomes isolated from plasma. Although the former is consistent with the finding that miR-34a is also downregulated in the human diabetic heart [52], we suggest that the latter may be released from other cells, as EXO-miR-34a showed a moderate negative correlation with cardiac output (Table 2, *p* = 0.093), suggesting a non-cardiac source, and miR-34a is upregulated in pancreatic and peripheral blood mononuclear cells (PBMCs) from DM patients [53,54].

Circulating miRNAs have an important role in the diagnosis of HF [13,14,15] and can reveal the mechanisms of HF progression [10,11,12]. Clinical studies have shown the predictive and diagnostic value of circulating miRNAs, such as miR-21 [55], miR-30d [56], miR-126a [57,58] and miR-423 [59]. MiRNA-based technologies are being developed for the treatment of heart diseases, including cardiac fibrosis, hypertrophy and HF [48,60]. Targeted therapies using miR-1 [61], miR-30d [62], miR-208a [61], miR-483 [63], miR-499 [61] and miR-1202 [63] are therapeutically effective in patients with HF. Using different miRNA panels, as in our study, can begin to distinguish between those miRNAs associated with HFpEF and HFrEF [27], e.g., inhibition of miRNA-21 prevents the development of HFpEF and is associated with reduced expression of the anti-apoptotic gene Bcl-2 [64]. MiRNA-based HF therapy is feasible for both HFpEF and HFrEF. Cocktail therapies including multiple miRNAs and miRNA inhibitors may yield significant results.

EXO-miRNAs are also promising molecules for the diagnosis and treatment of HF [65,66]. Existing examples include: cardiac fibroblast-derived EXO-miR-21 targets cardiomyocytes and induces cardiac hypertrophy [67]; increased EXO-miR-29a in the marginal ischemic zone mediates anti-fibrotic effects and prevents ventricular dysfunction [68]; cardiomyocytes negatively regulate endothelial cell proliferation and migration via EXO-miRNA-320, and delivery of EXO-miR-320 inhibits the development of HF [69]; EXO-miR-425 and EXO-miR-744 reduce the angiotensin-induced synthesis of collagen and cellulose and inhibit myocardial remodeling, and their reduction is associated with increased expression of type 1 collagen and a-SMA, leading to activation of cardiac fibroblasts [70]; EXO-miR-92b are increased in the serum of patients with acute HF and negatively correlate with left ventricular ejection fraction; thus, the levels of EXO-miR-92b-5p can be used as a biomarker for the diagnosis of HFrEF [71].

There are some limitations worth mentioning. First, the sample size in the study was at least six rats because, according to our previous work, hemodynamic analysis requires a large sample size to ensure the accuracy and confidence of the experiment. Therefore, 20 rats/group were initially assigned to the hemodynamic experiments (Figure 2A,B and Figure 3). To ensure optimal input quality, we excluded hemolyzed plasma and poor-quality EXO-RNA (260/280 ratio <1.9 or >2.1). Thus, we had ten hearts (Figure 2C,D and Figure 4), nine plasma (Figure 1C–F) and six EXO-RNA samples. To maintain pairwise consistency, six hearts and six EXOs were used in Figure 5 and Figure 7 to study miRNA expression. Second, although the accuracy of miRNA quantification may be affected by PCR overamplification, the concomitant results in EXO make the observations consistent. Finally, as a result of this study, candidate biomarkers were identified in rodent model. Regarding clinical patient data, we are collecting plasma samples from diabetic HFpEF patients. Exosomal miR-30d-5p and miR-126a-5p levels will be evaluated to confirm their clinical significance and will be addressed in future studies.

## 4. Materials and Methods

### 4.1. Experimental Animals

Sprague–Dawley (SD) male rats (8 weeks old, 250–300 g) were purchased from Biolasco (Taipei, Taiwan). Rats were randomly divided into two groups, the control group and the DM group. All animals were housed in an AAALAC-certified animal center at Chang Gung University (CGU) with ad libitum access to food (Labdiet 5001) and water. Rats were fasted and anesthetized by injection of Zoletil (50 mg/kg) + Rompun (10 mg/kg). Rats were then injected with fresh streptozocin (STZ, 65 mg/kg) and saline to induce DM and control rats. Animals exhibiting hyperglycemia (>300 mg/dL), polyphagia, polyuria and polydipsia were classified as induced diabetes. The sample size in the study was at least 6 (*n* = 6) to ensure experimental confidence. No blinding was performed, as the investigators assumed the responsibility for STZ injection and animal care. No animals were excluded except for those in which surgery-related deaths occurred. Animal studies were reported according to ARRIVE guidelines.

### 4.2. Biochemical Analysis

Blood glucose was measured using Touch Sure Step strips (Life Scan, Johnson and Johnson, Milpitas, CA, USA). Total cholesterol, triglyceride (TG), and non-esterified fatty acid (NEFA) levels were measured with commercially available kits (Cholesterol-CH7945; TG-TR313 and NEFA-FA115, Randox Laboratories, Antrim, UK). Plasma insulin levels were measured with an ELISA kit (Mercodia 10-1250-01; Uppsala, Sweden).

### 4.3. Mean Blood Pressure (MBP) and Hemodynamic Measurements

MBP and hemodynamic parameters were measured as previously described [24,72]. Briefly, control and DM rats were anesthetized with Zoletil (50 mg/kg) + Rompun (10 mg/kg). A pressure-volume (P-V) catheter (SPR-838; Millar Instruments, Houston, TX, USA) was inserted into the right common carotid artery to measure MBP. The microtip of the P-V catheter was then advanced into the left ventricle. After stabilization for 20 min, cardiac contractility was measured and recorded using an ARIA P-V conductance system at a sampling rate of 1 kHz (1000 samples/s) (Millar Instruments) coupled to a Powerlab/4SP analog-to-digital converter (AD Instruments, Mountain View, CA, USA). The cardiac P-V analysis program was used to analyze cardiac contractility (PVAN3.2, Millar Instruments).

### 4.4. Histology

Heart tissues were fixed in phosphate-buffered saline with 10% paraformaldehyde. The fixed heart tissues were sent to the Taipei Institute of Pathology for histological sectioning. Hematoxylin and eosin (H&E) and Masson’s trichrome staining were used to examine the cardiomyocyte morphology and tissue fibrosis in the control and DM groups.

### 4.5. Western Blotting

Heart tissue lysates were extracted by protease inhibitor (Bionovas Cat#FC007-0001, Toronto, ON, Canada) containing RIPA lysis buffer (Thermo Cat#89901, Rockford, IL, USA). Antibodies against COL1A2 (Bioworld Technology Cat#BS1530, St. Louis Park, MN, USA, RRID: AB_1662101) and tubulin (Cell signal Cat#2146, Danvers, MA, USA, RRID: AB_2210545) were diluted in TBST at a ratio of 1:1000. PVDF membranes were incubated with horseradish peroxidase (HRP)-conjugated secondary antibody (Thermo Cat#31460, Rockford, IL, USA, RRID: AB_228341) and subjected to chemiluminescence detection (Biorad, ChemiDoc Touch Imaging System, Hercules, CA, USA). Blotting results were quantified using ImageJ (ImageJ, RRID: SCR_003070).

### 4.6. Isolation of Plasma Exosomes

Plasma EXO was isolated by a serial centrifugation process. Briefly, the heparinized plasma was diluted 6-fold with PBS to reduce the viscosity of the plasma. Dead cells and cell debris were removed from the plasma by centrifugation at 13,200× *g* for 30 min at 4 °C. The supernatant was filtered through a 0.2 μm filter to remove non-EXO particles. Samples were then ultracentrifuged at 120,000× *g* for 70 min at 4 °C (Beckman Optima L-90K with a 70Ti rotor). EXO pellets were washed with PBS and recollected by centrifugation at 120,000× *g* for 70 min at 4 °C. EXO was suspended with PBS containing proteinase inhibitors and stored at −80 °C for further studies.

### 4.7. Nanoparticle Tracking Analysis (NTA)

The size and concentration of EXO in plasma were analyzed using an NTA system (NanoSight NS300, Malvern, UK). Briefly, EXO samples were diluted in sterilized deionized water at a ratio of 1:100, and their size distribution and concentration were measured based on the Brownian motion of vesicles. EXO was visualized and recorded with a charge-coupled device (CCD) camera (3 times, 60 s/time). Data were analyzed with NTA 3.0 software (Malvern Instruments, Malvern, UK).

### 4.8. Transmission Electron Microscope (TEM)

Four microliters of isolated extracellular vesicles were loaded onto a glow discharge carbon film supported copper grids (EMS CF-200-Cu) for 1 min and then washed twice with water. After removing excess liquid with filter paper, the grids were stained with 2% uranyl acetate for 1 min and then air dried. Micrographs were obtained in a JEM-1230 transmission electron microscopy (JEOL) at 100 kV with a Gatan Model 832 digital camera.

### 4.9. Real-Time Quantitative Polymerase Chain Reaction (RT-qPCR)

Total RNA from the heart was extracted using a TOOLSmart RNA extractor (DPT-BD24, BioTools Co., Ltd., New Taipei City, Taiwan). Myocardial and exosomal miRNAs were extracted using the miRNeasy kit (Qiagen, 1038703, Hilden, Germany). Prior to cDNA synthesis, DNAse I (Invitrogen, 18068-015, Carlsbad, CA, USA) was used to avoid genomic DNA contamination. cDNA synthesis was performed using the Gscript First-Strand Synthesis kit (GeneDirex, MB-305-0050, Taoyuan, Taiwan) according to the manufacturer’s protocol. In addition, cDNA synthesis of miRNA was repeated for 50 cycles to increase the yield of miRNA cDNA. RT-qPCR or stem-loop RT-qPCR quantification of cDNA levels of mRNAs or miRNAs of interest was performed using the 7500 Fast Real-Time PCR System (Applied Biosystems, Foster City, CA, USA). Internal controls were 18S ribosomal RNA and RNU6 (RNA, U6 small nuclear 1) to normalize specific genes and miRNAs, and 2^−ΔΔCT^ calculations were performed to obtain results. All primers are shown in Tables S1 and S2.

### 4.10. Statistical Analysis

The sample sizes of the experiments were empirically determined based on relevant research experience and are listed in the corresponding figure captions. Statistical analyses (GraphPad Prism Software 8, San Diego, CA, USA, RRID: SCR_002798) were performed for each group size of at least *n* = 5 independent samples/individuals. Data are expressed as mean ± standard error. Significance was analyzed using Student’s unpaired *t*-test. A two-tailed *p* < 0.05 was considered significant. Pearson correlation coefficients between 0.8 and 1.0 indicated a very strong correlation; between 0.6 and 0.8 indicated a strong correlation; between 0.4 and 0.6 indicated moderate correlation; between 0.2 and 0.4 indicated weak correlation; between 0.0 and 0.2 indicated very weak correlation, as described elsewhere [73].

## Figures and Tables

**Table 1:** Pearson correlation between cardiac miRNAs and cardiac output.

	Description	r	*p* (Two-Tailed)
miR-1-3p	Very strong	0.8384	<0.001
miR-21-5p	Strong	0.7027	0.011
miR-29a-5p	Very strong	0.8648	<0.001
miR-30d-5p	Very strong	0.8654	<0.001
miR-34a-5p	Very strong	0.8249	<0.001
miR-126a-5p	Strong	0.7905	0.002
miR-143-3p	Moderate	0.582	0.047
miR-145-5p	Strong	0.7029	0.011
miR-195-p	Strong	0.6016	0.039
miR-206-3p	Strong	0.7557	0.004
miR-320-3p	Moderate	0.5915	0.043
miR-378-3p	Moderate	0.5624	0.057

**Table 2:** Pearson correlation between EXO-miRNAs and cardiac output.

	Description	r	*p* (Two-Tailed)
miR-1-3p	Very weak	0.1388	0.667
miR-21-5p	Moderate	0.4645	0.128
miR-29a-5p	Very weak	0.1724	0.592
miR-30d-5p	Strong	0.6317	0.028
miR-34a-5p	Moderate	−0.5058	0.093
miR-126a-5p	Strong	0.6375	0.026
miR-143-3p	Weak	0.3964	0.202
miR-145-5p	Moderate	0.4032	0.194
miR-195-5p	Moderate	0.4266	0.167
miR-206-3p	Weak	0.3795	0.224
miR-320-3p	Moderate	0.4541	0.138
miR-378-3p	Strong	0.6412	0.025

## Data Availability

All data in this study are available from the corresponding author upon reasonable request.

## Supplementary Materials

The following supporting information can be downloaded at: https://www.mdpi.com/article/10.3390/ijms23147514/s1.
