# Supplementary material for: Exosomal microRNAs miR-30d-5p and miR-126a-5p Are Associated with Heart Failure with Preserved Ejection Fraction in STZ-Induced Type 1 Diabetic Rats
Source: Int J Mol Sci. 2022 Jul 6;23(14):7514. doi: 10.3390/ijms23147514 (PMC9318774; doi:10.3390/ijms23147514)
Supplement: Supplementary file 1 [file ijms-23-07514-s001.zip › ijms-1787384-supplementary.pdf]

Table S1. Primer sets for RT-qPCR

Gene	Accession number	Forward	Reverse	Amplicon length (bp)
Myh6	NM_012612.2	GGAAGTCAACCCGTCTCAGA	TGGGCTCCAATCCTGTCAAT	168
Myh7	NM_031545.1	AGTCCTAGCCAGTCTCCAGA	GTCTCTCCTGGATCCGGAAG	172
Nppa	NM_017239.2	AAGCCCACTTCTCTCTGGTC	CCTTTGCCTTTCCCACTGTC	190
Nppb	NM_017240.2	CCAGTCCCGAGGTGTACTTT	TCCTCCTTCATGTTGGCCAT	195
Rn18s	NR_046237	GCAATTATTCCCCATGAACG	GGCCTCACTAAACCATCCAA	123

Table S2. Primer sets for stem-loop RT-qPCR

Assay	Accession number	Reverse transcription stem loop primer	PCR forward primer	Universal PCR reverse primer
miR-1-3p	MIMAT0003125	CTCAACTGGTGTCGTGGAGTCGGCAATTCAGTTGAGATACACACTT	CGGTGGAATGTAAAGAAGTGTGTAT	CTCAACTGGTGTCGTGGAGT
miR-21-5p	MIMAT0000790	CTCAACTGGTGTCGTGGAGTCGGCAATTCAGTTGAGTCAACATCAG	CGGTAGCTTATCAGACTGATGTTGA	
miR-29a-5p	MIMAT0004718	CTCAACTGGTGTCGTGGAGTCGGCAATTCAGTTGAGCTGAACACCA	CGGACTGATTTCTTTTGGTGTTCAG	
miR-30d-5p	MIMAT0000807	CTCAACTGGTGTCGTGGAGTCGGCAATTCAGTTGAGCTTCCAGTCG	TGTAAACATCCCCGACTGGAAG	
miR-34a-5p	MIMAT0000815	CTCAACTGGTGTCGTGGAGTCGGCAATTCAGTTGAGACAACCAGCT	TGGCAGTGTCTTAGCTGGTTGT	
miR-126a-5p	MIMAT0000831	CTCAACTGGTGTCGTGGAGTCGGCAATTCAGTTGAGCGCGTACCAA	CGGCATTATTACTTTTGGTACGCG	
miR-143-3p	MIMAT0000849	CTCAACTGGTGTCGTGGAGTCGGCAATTCAGTTGAGTGAGCTACAG	TGAGATGAAGCACTGTAGCTCA	
miR-145-5p	MIMAT0000851	CTCAACTGGTGTCGTGGAGTCGGCAATTCAGTTGAGAGGGATTCCT	GTCCAGTTTTCCCAGGAATCCCT	
miR-195-5p	MIMAT0000870	CTCAACTGGTGTCGTGGAGTCGGCAATTCAGTTGAGGCCAATATTT	CGGTAGCAGCACAGAAATATTGGC	
miR-206-3p	MIMAT0000879	CTCAACTGGTGTCGTGGAGTCGGCAATTCAGTTGAGCCACACACTT	TGGAATGTAAGGAAGTGTGTGG	
miR-320-3p	MIMAT0000903	CTCAACTGGTGTCGTGGAGTCGGCAATTCAGTTGAGTCGCCCTCTC	AAAAGCTGGGTTGAGAGGGCGA	
miR-378a-3p	MIMAT0003379	CTCAACTGGTGTCGTGGAGTCGGCAATTCAGTTGAGCCTTCTGACT	ACTGGACTTGGAGTCAGAAGG	
RNU6	NR_004394.1	CTCAACTGGTGTCGTGGAGTCGGCAATTCAGTTGAGAAAATATGGAACG	CAAATTCGTGAAGCGTTCCA	

Figure S1

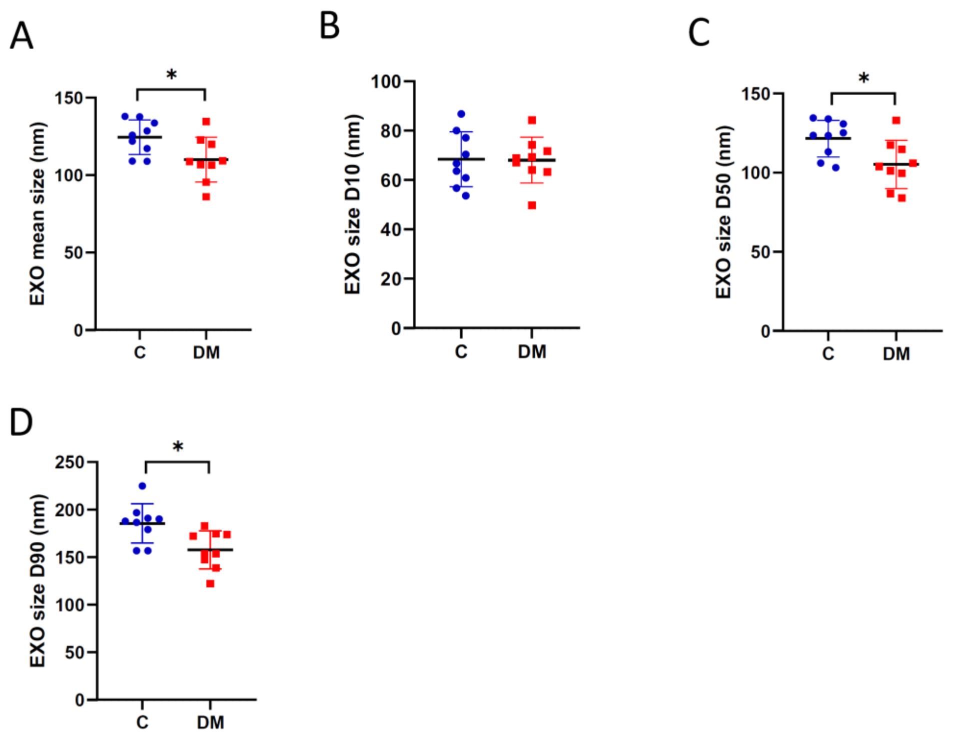

Figure S1. Size distribution of EXOs in control and DM plasma. The size-concentration distribution of EXOs was analyzed by nanoparticle tracking analysis (NTA). (A) Mean size, (B) size D10, (C) size D50, (D) size D90. Data are presented as mean ± SEM (n=9). P-values were determined by Student's *t*-test.

Figure S2

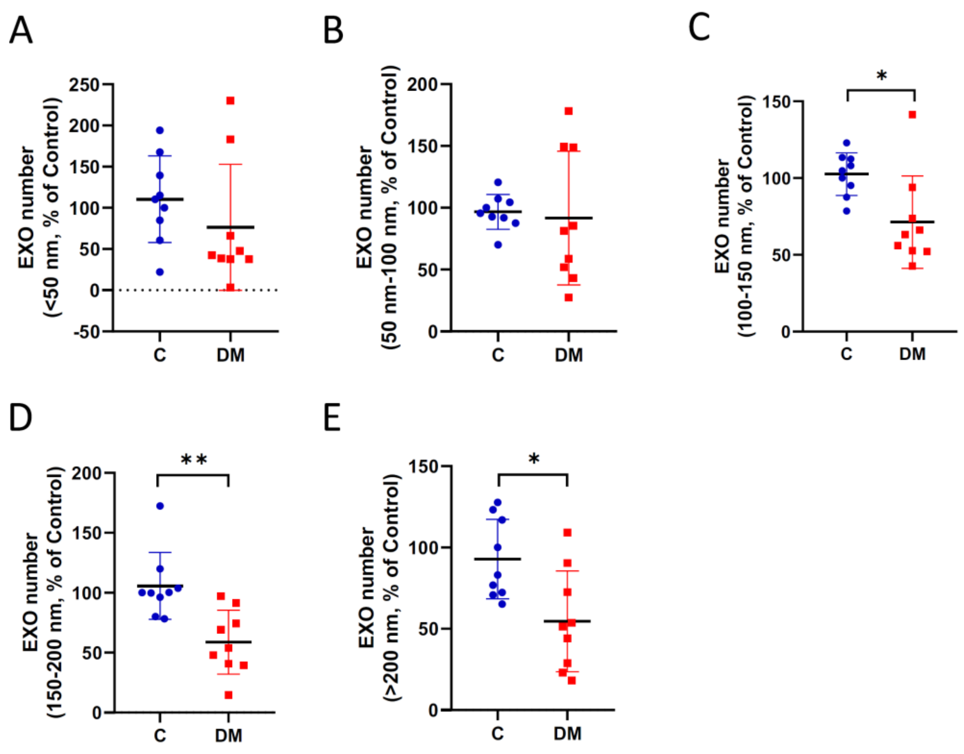

Figure S2. EXO number in control and DM plasma. The size-concentration distribution of EXOs was analyzed by nanoparticle tracking analysis (NTA). (A) EXO number of the size <50 nm, (B) 50 nm-100 nm, (C) 100-150 nm, (D) 150-200 nm and (E) >200 nm) Data are presented as mean ± SEM (n=9). P-values were determined by Student's *t*-test.
